# Synthesis of Some New Tetrahydropyrimidine Derivatives as Possible Antibacterial Agents

**Published:** 2017

**Authors:** Naser Foroughifar, Somayeh Karimi Beromi, Hoda Pasdar, Masoumeh Shahi

**Affiliations:** *Department of Chemistry, Tehran North Branch, Islamic Azad University, Tehran, Iran.*

**Keywords:** Tetrahydropyrimidine, antimicrobial activity, Biginelli reaction

## Abstract

Heterocyclic compounds containing a pyrimidine nucleus are of special interests thanks to their applications in medicinal chemistry as they are the basic skeleton of several bioactive compounds such as antifungal, antibacterial, antitumor and antitubercular. As a part of our research in the synthesis of pyrimidine derivatives containing biological activities, some new tetrahydropyrimidine derivatives (1-10) were synthesized via Biginelli reaction using HCl or DABCO as a catalyst with good yields. All structures of products were confirmed by IR, ^1^H NMR and ^13^C NMR spectroscopy. The antibacterial activity of some synthesized compounds was investigated against *Staphylococcus* *aureus *(ATCC 6538), *Staphylococcus epidermidis *(ATCC 12228)*,* *Bacillus cereus *(ATCC14579)*, Esherichia coli *(ATCC 25922), *Klebsiella pneumonia *(ATCC 13883) and *Pseudomonas aeruginosa *(PAO1) bacteria. Some of these compounds such as 8 and 10 exhibited a good to significant antibacterial activity.

## Introduction

Tetrahydroprymidines and their derivatives have recently attracted considerable interest thanks to their pharmacological activities such as anticancer (1), antiviral (2), calcium channel modulation (3) and antibacterial activity (4-6). The Biginelli reaction is one of the simple and direct methods for the synthesis of tetrahydropyrimidines, originally reported by Biginelli (7). Regarding the importance of the Biginelli reaction products, much work on improving the yield and reaction conditions has been actively pursued. For example, using Lewis acids as a catalyst such as Cu(OTf)_2_ (8), Yb(OTf)_3_ (9), Triethylammonium hydrogen sulfate (10), BiCl_3_ (11) and Mn(OAc)_3_.2H_2_O (12) instead of acidic reagents significantly improved the reaction output with reduced reaction times. The polymer-supported, resin-bound isothiourea (13), polymer nanocomposite (14) and various other catalysts (15, 16) have been used for synthesis of Biginelli products. In general terms, this report is going to describe the synthesis of new tetrahydropyrimidine derivatives via the Biginelli reaction using HCl or DABCO as a catalyst in ethanol. Biological activities of synthesis compounds were tested against gram-positive and gram-negative bacteria.

## Experimental

Melting points were determined with an Electrothermal digital apparatus and were uncorrected. IR spectra were obtained on a Galaxy Series FT-IR 5000 spectrophotometer in KBr. NMR spectra were recorded on a Brucker 500 and 300 MHz spectrometer, chemical shifts were given in ppm in DMSO-d_6_ using TMS as an internal standard.

**Scheme 1:**
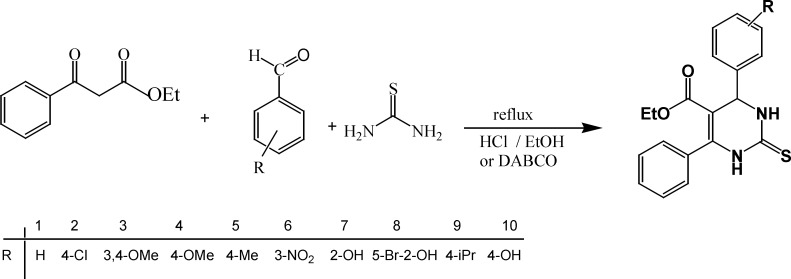
The synthetic pathway for preparation of tetrahydropyrimidine derivatives 1-10

**Table 1:** Antibacterial activity of some synthesized compounds (inhibition zones, mm).

**Comp. No**	***S. aureus***	***S*** ***. *** ***epidermidis***	***Bacillus cereus***	***K. pneumoniae***	***E. Coli***	***P. aeruginosa***
1	20	-	-	-	-	-
2	15	30	-	-	-	15
3	14	-	-	-	-	-
4	10	18	-	-	-	5
6	15	-	-	10	-	-
8	45	58	32	45	39	50
9	15	25	-	-	-	-
10	14	22	mutation	15	-	-
*Cephalexin* *	34	35	-	29	26	-

Dimethyl sulfoxide (DMSO) only, control for compounds and references.

* Reference compound

**Table 2:** MIC values of some synthesized compounds

**Comp. No**	**MIC (μg.mL** ^-1^ **)**					
	***S. aureus***	***S. epidermidis***	***Bacillus cereus***	***K. pneumoniae***	***E. Coli***	***P.aeruginosa***
1	37	25	NP	95	120	100
2	45	25	120	80	105	50
3	45	95	NP	100	NP	65
4	20	15	110	100	100	120
6	45	80	NP	50	130	85
8	15	15	25	15	15	15
9	45	30	NP	95	110	70
10	45	15	NP	50	110	NP
*Cephalexin* ^*^	10	15	50	23	15	46

*NP*: not performed

* Reference compound

**Table 3:** Characterization data of tetrahydropyrimidine derivatives 1-10.

**m.p (** ^o^ **C)**	**Yield (%)** *		**Time (h)**		**Ar**	**Product**
	**Catalyzed by DABCO**	**Catalyzed by HCl**	**Catalyzed by DABCO**	**Catalyzed by HCl**		
197-200	70	45	4	7	C_6_H_5_	1
178-180	72	63	5	7	4-Cl-C_6_H_4_	2
213-216	81	63	3	7	3,4-OMe_2_-C_6_H_3_	3
202-204	81	63	3	7	4-OMe-C_6_H_4_	4
179-182	88	53	4	7	4-Me-C_6_H_4_	5
226-228	71	52	4	7	3-NO_2_-C_6_H_4_	6
243-245	81	50	3	7	2-OH-C_6_H_4_	7
127-129	79	50	4	7	5-Br-2-OH-C_6_H_3_	8
211-213	70	62	4	7	4-ipr--C_6_H_4_	9
218-220	74	54	3	7	4-OH-C_6_H_4_	10

* Reported yields are after recrystallization

*General procedure for synthesis of tetrahydropyrimidine derivatives* (1-10)

A mixture of an ethyl benzoylacetate (1 mmol), aromatic aldehyde (1 mmol), thiourea (1 mmol) and an amount of concentration Hydrochloric acid or DABCO (0.1 mmol) in ethanol (15 mL) were heated under reflux for an appropriate time (Table 3). The progress of the reaction was monitored by TLC (Thin-Layer Chromatography) using water-ethanol (1:1) as an eluent and after competition, the reaction mixture was cooled at room temperature. The remaining solid was filtered, washed with water and ethanol and it was consequently dried and recrystallized using ethanol.

*5-Ethoxycarbonyl-4,6-diphenyl-1,2,3,4-tetrahydropyrimidine-2-thione *(1): IR (KBr) ν_max_ (cm^-1^): 3386 (NH), 3365 and 2937 (CH), 1676 (C=O), 1567 (C=S), 1369 (C=C) and 1336 (C-O). ^1^H NMR (DMSO-d6) δ ppm: 10.53 (s, 1H, NH); 9.80 (s, 1H, NH); 7.14-7.43 (m, 10H, Ar-H); 5.27 (s, 1H, H(4)); 3.71-3.79 (q, J=7.5 Hz, 2H, CH_2_) and 0.71-0.76 (t, J=7.5 Hz, 3H, CH_3_). ^13^C NMR (DMSO-d6) δ ppm: 13.36, 54.09, 59.51, 101.77, 126.43, 127.74, 127.86, 128.70, 128.74, 129.16, 134.00, 143.02, 145.90, 164.92 and 174.50.

*5-Ethoxycarbonyl-4-(4-chlorophenyl)-6-phenyl-1,2,3,4-tetrahydropyrimidine-2-thione *(2)*: *IR (KBr) ν_max_ (cm^-1^): 3304 (NH), 3158 and 2981 (CH), 1735 (C=O), 1589 (C=S), 1469 (C=C), 1432 (C-O) and 724 (C-Cl). ^1^H NMR (DMSO-d6) δ ppm: 10.71 (s, 1H, NH); 9.90 (s, 1H, NH); 7.38-8.22 (m, 9H, Ar-H); 5.41 (s, 1H, H(4)); 3.71-3.78 (q, J=7.0 Hz, 2H, CH_2_) and 0.70-0.74 (t, J=7.0 Hz, 3H, CH_3_). ^13^C NMR (DMSO-d6) δ ppm: 13.33, 53.44, 59.71, 100.86, 121.21, 122.92, 127.81, 128.71, 129.39, 130.63, 133.07, 133.66, 144.99, 146.75, 147.97, 164.81 and 174.81.

*5-Ethoxycarbonyl-4-(3,4-dimethoxyphenyl)-6-phenyl-1,2,3,4-tetrahydropyrimidine-2-thione *(3)*: *IR (KBr) ν_max_ (cm^-1^): 3415 (NH), 3060 and 2925 (CH), 1735 (C=O), 1570 (C=S), 1493 (C=C), 1455 (C-O) and 1291 (C-C). ^1^H NMR (DMSO-d6) δ ppm: 10.48 (s, 1H, NH); 9.74 (s, 1H, NH); 6.87-7.43 (m, 8H, Ar-H); 5.22 (s, 1H, H(4)); 3.75-3.80 (q, J=7.1 Hz, 2H, CH_2_); 3.73, 3.71 (both s, 3H each, 2 O-CH_3_) and 0.73-0.78 (t, J=7.2 Hz, 3H, CH_3_). ^13^C NMR (DMSO-d6) δ ppm: 13.80, 54.11, 55.85, 59.88, 102.33, 110.83, 121.21, 122.92, 127.81, 128.71, 129.39, 130.63, 133.07, 133.66, 144.99, 146.75, 149.11, 165.34 and 174.81.

*5-Ethoxycarbonyl-4-(4-methoxyphenyl)-6-phenyl-1,2,3,4-tetrahydropyrimidine-2-thione *(4)*: *IR (KBr) ν_max_ (cm^-1^): 3165 (NH), 2975 and 2836 (CH), 1694 (C=O), 1599 (C=S), 1463 (C=C), 1368 (C-O) and 1249 (C-C).^ 1^H NMR (DMSO-d6) δ ppm: 10.42 (s, 1H, NH); 9.87 (s, 1H, NH); 6.65-7.96 (m, 9H, Ar-H); 5.22 (s, 1H, H(4)); 3.74 (s, 3H, O-CH_3_); 3.87-3.71 (q, J=7.1 Hz, 2H, CH_2_) and 0.71-0.74 (t, J=7.1 Hz, 3H, CH_3_). ^13^C NMR (DMSO-d6) δ ppm: 14.21, 54.11, 55.99, 60.30, 102.97, 114.88, 128.54, 128.56, 129.00, 129.50, 129.3, 130.52, 132.67, 134.95, 136.41, 159.17, 165.78 and 175.17.

*5-Ethoxycarbonyl-4-(4-methylphenyl)-6-phenyl-1,2,3,4-tetrahydropyrimidine-2-thione *(5)*: *IR (KBr) ν_max_ (cm^-1^): 3367 (NH), 3108 and 2975 (CH), 1698 (C=O), 1571 (C=S), 1464 (C=C), 1206 (C-O) and 1097 (C-C). ^1^H NMR (DMSO-d6) δ ppm: 10.44 (s, 1H, NH); 9.70 (s, 1H, NH); 6.86-7.29 (m, 9H, Ar-H); 5.21 (s, 1H, H(4)); 3.69-3.78 (q, J=7.0 Hz, 2H, CH_2_); 2.28 (s, 3H, C(4)-p-CH_3_-Phenyl) and 0.69-0.74 (t, J=7.1 Hz, 3H, CH_3_).

*5-Ethoxycarbonyl-4-(3-nitrophenyl)-6-phenyl-1,2,3,4-tetrahydropyrimidine-2-thione *(6)*: *IR (KBr) ν_max_ (cm^-1^): 3421 (NH), 3086 and 2927 (CH), 1727 (C=O), 1583 (C=S), 1476 (C=C), 1445 (C-O) and 1293 (C-C). ^1^H NMR (DMSO-d6) δ ppm: 8.35 (s, 1H, NH); 8.05 (s, 1H, NH); 7.56-7.92 (m, 9H, Ar-H); 5.51 (s, 1H, H(4)); 3.57-3.30 (q, J=7.1 Hz, 2H, CH_2_) and 0.98-1.03 (t, J=7.1 Hz, 3H, CH_3_).

*5-Ethoxycarbonyl-4-(2-hydroxyphenyl)-6-phenyl-1,2,3,4-tetrahydropyrimidine-2-thione *(7)*: *IR (KBr) ν_max_ (cm^-1^): 3381 (NH), 3089 and 2983 (CH), 1693 (C=O), 1580 (C=S), 1491 (C=C), 1459 (C-O) and 1260 (C-C). ^1^H NMR (DMSO-d6) δ ppm: 12.25 (s, 1H, OH); 8.55 (s, 1H, NH); 8.26 (s, 1H, NH); 7.51-8.21 (m, 9H, Ar-H); 5.70 (s, 1H, H(4)); 3.54-3.31 (q, J=7.2 Hz, 2H, CH_2_) and 0.78-0.93 (t, J=7.1 Hz, 3H, CH_3_).

*5-Ethoxycarbonyl-4-(5-bromo-2-hydroxyphenyl)-6-phenyl-1,2,3,4-tetrahydropyrimidine-2-thione *(8)*: *IR (KBr) ν_max_ (cm^-1^): 3526 (OH), 3304 (NH), 3166 and 2978 (CH), 1723 (C=O), 1576 (C=S), 1489 (C=C), 1394 (C-O), 1289 (C-C) and 823 (C-Br). ^1^H NMR (DMSO-d6) δ ppm: 10.19 (s, 1H, OH); 7.68 (s, 1H, NH); 7.62 (s, 1H, NH); 6.93-7.63 (m, 8H, Ar-H); 5.25 (s, 1H, H(4)); 3.67-3.70 (q, J=7.1 Hz, 2H, CH_2_) and 0.95-1.01 (t, J=7.0 Hz, 3H, CH_3_).

*5-Ethoxycarbonyl-4-(4-isopropylphenyl)-6-phenyl-1,2,3,4-tetrahydropyrimidine-2-thione *(9)*: *IR (KBr) ν_max_ (cm^-1^): 3422 (NH), 3186 and 2925 (CH), 1629 (C=O), 1484 (C=S), 1386 (C=C), 1245 (C-O) and 1081 (C-C). ^1^H NMR (DMSO-d6) δ ppm: 10.49 (s, 1H, NH); 9.75 (s, 1H, NH); 7.29-7.43 (m, 9H, Ar-H); 5.23 (s, 1H, H(4)); 3.37-3.79 (q, J=7.5 Hz, 2H, CH_2_), 1.19-1.22 (m, 7H, H iPr) and 0.70-0.79 (t, J=7.5 Hz, 3H, CH_3_).

*5-Ethoxycarbonyl-4-(4-hydroxyphenyl)-6-phenyl-1,2,3,4-tetrahydropyrimidine-2-thione *(10)*: *IR (KBr) ν_max_ (cm^-1^): 3489 (OH), 3318 (NH), 3178 and 3000 (CH), 1683 (C=O), 1566 (C=S), 1462 (C=C), 1370 (C-O) and 1333 (C-C).

*Antibacterial Activity*

To examine the antibacterial activity of some synthesized compounds, three gram negative bacteria: *Esherichia coli* (ATCC 25922), *Klebsiella pneumoniae* (ATCC 13883) and *Pseudomonas aeruginosa* (PAO1) and three gram positive bacteria: *Staphylococcus aureus* (ATCC 6538), *Staphylococcus epidermidis* (ATCC 12228), *Bacillus cereus* (ATCC 14579) were selected and tested by the disc diffusion method (17) using Mueller–Hinton agar against. Cephalexin was used as the standard. Normal saline was used for preparation of inoculants having turbidity equal to 0.5 McFarland standards. Tested compounds were dissolved in dimethyl sulfoxide (DMSO) for the preparation of stock solution. The solvent control was included, although no antibacterial activity has been noted. Culture was carried out with sterile swab and microtube suspension was cultured for 24 h and then inoculated onto Mueller Hinton agar. Blank discs with a diameter of 6 mm and containing 30 µg of the concentration of these compounds (1-10) were placed on Muller Hinton agar medium. After 24 h incubation at 37 °C, zones of growth inhibition were measured. Disks containing 10 µg of dimethyl sulfoxide were used as the negative control. Each concentration was repeated 4 times for each of the bacteria and the average results of inhibitory effects are illustrated in Table 1.

Determination of the minimum inhibitory concentration (MIC) values for some synthesized compounds against six microorganisms was carried out using disc diffusion method (18). In this method, concentration of 10, 20, 30, 50, ……., 150 µg/mL were used for all bacteria per disc and there were incubated at 37 °C for 24 h. MIC value was defined as lowest concentration of compound for inhibition growth of the tested bacteria. The results are shown in Table 2.

## Results and Discussion

The benzaldehyd derivatives with substitution in aromatic ring with 4-chloro, 3,4-dimethox, 4-methoxy, 4-methyl, 3-nitro, 2-hydroxy, 5-bromo-2-hydroxy, 4-isopropyl and 4-hydroxy groups were reacted with thiourea and ethyl benzoylacetate in the presence of HCl or DABCO as a catalyst under reflux condition to prepare a series of tetrahydropyrimidine derivatives 1-10 (scheme1, Table 3). According to Table 3, the best results were obtained in the presence of DABCO as a catalyst where the products were achieved with high yields and shorter reaction times. 

The IR spectra of tetrahydropyrimidine derivatives 1-10 exhibited absorption bands at 1570 and 1590 cm^-1^ relating to C=S and C=O, respectively. The broad absorption band for stretching vibration of NH groups was detected in the region 3100-3360 cm^-1^. In ^1^H NMR spectra, all of the products 1-10 showed a singlet peak at about 5.2-5.4 ppm for H-4. Two singlet peaks for NH groups in pyrimidine ring were observed at about 10.4-10.7 and 9.7-9.9 ppm, which disappearing upon D_2_O addition. The ^13^C NMR spectra of these compounds showed a signal at about 164.8-165.7 for C=S and a signal at about 174.5-175.1 ppm for C=O group.

Antibacterial activities of compounds 1- 4, 6 and 8- 10 were measured on three gram negative bacteria (E. coli, K. pneumoniae and P. aeruginosa) and three gram positive bacteria (S. aureus, S. epidermidis and B. cereus) by disc diffusion method and the minimum inhibitory concentration (MIC) in-vitro. Cephalexin was used as the standard antibacterial agent. The results of bioassay are given in Tables 1 and 2. As shown in Table 2, these compounds exhibited good inhibitory activity against S. aureus with MIC values about 15-45 µg/mL. Compounds 1, 2, 4 and 8-10 exhibited remarkable activity against S. epidermidis. Except for compound 8, the other compounds did not show any inhibitory activity against E. coli, K. pneumoniae and B. cereus. Compounds 2 and 8 showed considerable inhibitory activity against P. aeruginosa but the other compounds did not show any activity against P. aeruginosa. Generally, Compound 8, which contains the 5-bromo-2-hydrophenyl moiety, indicates more inhibitory activity (15-25 µg/mL) against all organist tests, in comparison to Cephalexin which is a well-known antimicrobial drug.
